# Altered Resting-State Brain Activity and Connectivity in Depressed Parkinson’s Disease

**DOI:** 10.1371/journal.pone.0131133

**Published:** 2015-07-06

**Authors:** Xiao Hu, Xiaopeng Song, Erfeng Li, Jiajia Liu, Yonggui Yuan, Weiguo Liu, Yijun Liu

**Affiliations:** 1 Department of Neurology, Affiliated Brain Hospital of Nanjing Medical University, Nanjing, 210029, China; 2 Department of Biomedical Engineering, College of Engineering, Peking University, Beijing, 10087, China; 3 Department of Psychiatry and Psychosomatics, Affiliated ZhongDa Hospital of Southeast University, The Institute of Neuropsychiatry of Southeast University, Nanjing, 210009, China; Wake Forest School of Medicine, UNITED STATES

## Abstract

Depressive symptoms are common in Parkinson’s disease (PD), but the neurophysiological mechanisms of depression in PD are poorly understood. The current study attempted to examine disrupted spontaneous local brain activities and functional connectivities that underlie the depression in PD. We recruited a total of 20 depressed PD patients (DPD), 40 non-depressed PD patients (NDPD) and 43 matched healthy controls (HC). All the subjects underwent neuropsychological tests and resting-state fMRI scanning. The between-group differences in the amplitude of low frequency fluctuations (ALFF) of BOLD signals were examined using post-hoc tests after the analysis of covariance. Compared with the NDPD and HC, the DPD group showed significantly increased ALFF in the left median cingulated cortex (MCC). The functional connectivity (FC) between left MCC and all the other voxels in the brain were then calculated. Compared with the HC and NDPD group, the DPD patients showed stronger FC between the left MCC and some of the major nodes of the default mode network (DMN), including the post cingulated cortex/precuneus, medial prefrontal cortex, inferior frontal gyrus, and cerebellum. Correlation analysis revealed that both the ALFF values in the left MCC and the FC between the left MCC and the nodes of DMN were significantly correlated with the Hamilton Depression Rating Scale score. Moreover, higher local activities in the left MCC were associated with increased functional connections between the MCC and the nodes of DMN in PD. These abnormal activities and connectivities of the limbic-cortical circuit may indicate impaired high-order cortical control or uncontrol of negative mood in DPD, which suggested a possible neural mechanism of the depression in PD.

## Introduction

In addition to the motor symptoms (tremor, rigidity, bradykinesia, and loss of postural reflexes), the non-motor symptoms such as psychiatric symptoms are common in Parkinson’s disease (PD) [1]. Depression affects approximately 35% of PD patients[2], this rate is much higher than that in the general population or in other comparable chronic disabling diseases[3]. Depression is associated with increased severity of disability [4], it plays a significant role in PD patients’ poor sense of well-being[5] and shortens PD patients’ life expectancy[1]. However, the neural basis of depression in PD is poorly understood.

Resting-state fMRI (RS-fMRI) can examine spontaneous brain activities in vivo and has been widely used for investigating the long-term effects of neurodegenerative disease on the brain. The amplitude of low-frequency fluctuations (ALFF) and functional connectivity (FC) are two different RS-fMRI data analysis methods. The ALFF measures the amplitude of spontaneous, low-frequency(0.01~0.08 Hz) blood oxygen level dependent (BOLD) signal and can be used to characterize spontaneous neural activity at a local level, while the FC is indicated by the correlation of the temporal patterns of BOLD signals in two different brain regions[6], and can be used to characterize the functional relationship between two regions at a network level. Both methods have been widely used to detect abnormal local activity and global connectivity associated with some neuropsychiatric disorders, including Alzheimer’s disease[7], epilepsy [8], depression[9], and PD[10, 11].

Previous studies have shown that the dysfunction of prefrontal-limbic network was involved in depression[12], and it was hypothesized that the depressive PD (DPD) patients would also show a dysfunction of prefrontal-limbic network. Both Surdhar et al.[13] and Luo et al.[14] have reported abnormal prefrontal-limbic network activities in DPD patients. Our previous study also found abnormal corticolimbic networks in DPD patients[15]. However, because of the complexity and multidimensional causes of DPD together with variability between individuals, we thought that there should be some other regions involved in depression in PD. So, we choose the data-driven analysis to study our data without any hypothesis. In our current research, we identified ROIs with abnormal local spontaneous brain activity between DPD and NDPD, HC. We then used the ROIs as seeds to find out the network-level alterations in DPD patients. We also hypothesized that abnormal local activities should lead to global abnormalities at the network level in different neural circuits and resulted in depressive symptoms in PD.

## Materials and Methods

### Participants

The study was approved by the Medical Research Ethical Committee of Nanjing Brain Hospital. Written informed consents of all the subjects were obtained. Sixty individuals (36males) with idiopathic PD and 43 healthy controls (HC) (21 males) were recruited. All the participants were right-handed Han Chinese. All of the PD patients fulfilled the UK Parkinson’s Disease Society Brain Bank criteria for idiopathic PD [16]. Clinical tests and MRI scans were performed to exclude acute physical illness, primary neurological illness, and other major psychiatric illness, such as brain tumor, stroke or dementia. Any subjects with Mini-Mental State Examination (MMSE) scores <24 or the use of dopamine agonists were excluded, as were those who used antidepressants before the beginning of the study. Similar exclusion criteria were adopted for the HC group. The dopamine dosing was stable for at least 4 weeks before and during the study. The programs of participants’ recruitment and follow-up information have been described in our previous study[15].

### Neuropsychological measurements

For each PD patient, all psychometric and neurologic evaluations were conducted during a practically defined “on” state, i.e. without showing any typical motor symptoms, without medication for depression, before and during the MRI scan. Motor severity was assessed by the Hoehn and Yahr (H&Y) staging scale and the Unified Parkinson’s Disease Rating Scale motor part III (UPDRS III). All subjects performed the MMSE score and the 17-item Hamilton Depression Rating Scale (HDRS-17). First, DPD patients were diagnosed with the DSM-V criteria by an experienced psychiatrist. Second, the severity of depression in PD patients was assessed by the HDRS-17 and all DPD patients diagnosed by DSM-V criteria also had scores of HDRS-17 higher than 14 points[17].

### Image acquisition

All the patients were in the “on” state before and during MRI. MRI data were acquired using a Siemens 3.0-Tesla signal scanner (Siemens, Verio, Germany). Participants were instructed to remain as still as possible, close their eyes, remain awake and not to think of anything. Axial anatomical images were acquired using a T1-FLAIR sequence (TR/TE = 2530ms/3.34ms, flip angle = 7°, matrix = 256×192, FOV = 256mm×256mm, slice thickness/gap = 1.33 mm/0.5mm, 128 slices covered the whole brain) for image registration and functional localization. Functional images were subsequently collected in the same slice orientation with a gradient-recalled echo-planar imaging (GRE-EPI) pulse sequence (TR/TE = 2000ms/30 ms, flip angle = 90°, matrix = 64×64, FOV = 220 mm×220 mm, thickness/gap = 3.5 mm/0.6mm, in-plane resolution = 3.4 mm×3.4 mm, slices numbers = 31). 140 volumes were obtained in this acquisition sequence and each functional resting-state session lasted 280s.

### Data preprocessing

Functional images were preprocessed with Statistical Parametric Mapping software (SPM8, www.fil.ion.ucl.ac.uk/spm). For each subject, the first ten volumes of the scanned data were discarded. The remaining images were corrected by realignment account for head motion. Three subjects (2 HC and 1 NDPD) with head motion exceeding 2.5 mm maximum displacement in the translation or 2.5° of angular motion throughout the course of the scan were excluded from the following analysis. Individual 3D T1-weighted anatomical image was coregistered to functional images. The 3D T1-weighted anatomical images were segmented (grey matter, white matter, and cerebrospinal fluid). A nonlinear spatial deformation was then calculated from the grey matter images to a grey matter template in Montreal Neurological Institute space using 12 parameters affine linear transformation. This transformation was then applied to the functional images, which were resliced at a resolution of 3×3×3mm^3^, and spatially smoothed with a Gaussian kernel (full-width at half-maximum = 6×6×6mm^3)^. The resulting fMRI data were band-pass filtered (0.01 < *f* < 0.08 Hz). Any linear trend was removed. There is no significant difference of the mean head motion parameters among the three groups (*P* = 0.068). Six head motion parameters and the mean time series of global, white matter and cerebrospinal fluid signals were introduced as covariates into a random effects model to remove possible effects of head motion, global, white matter and cerebrospinal fluid signals on the results.

### Functional image analysis

The ALFF map of each subject was calculated and transformed to z-map with the REST software (http://www.resting-fmri.sourceforge.net). These regions with DPD group vs non-depressive PD (NDPD) group ALFF significantly differences were defined as ROIs. The left median cingulated cortex (MCC) was selected as seed region based on the ALFF findings. Voxel-wise FC analysis was performed by computing the temporal correlation between each ROI’s mean time course and each voxel’s time course in the brain. FC maps were transferred to Z-scores maps with Fisher’s r-to-z transformation. Therefore, a Z-score map of the entire brain was created for each ROI for each subject.

### Statistical Analysis

An analysis of covariance (ANCOVA) was performed on the ALFF maps to identify brain areas with significant differences among the three groups (voxel-level *p* < 0.01, cluster size > 918 mm^3^ /34 voxels, corresponding to a corrected *p* < 0.05 as determined by AlphaSim correction). These areas were then extracted as a mask. A two-sample post hoc *t*-test of the ALFF maps within this mask was performed between each pair of the three groups (DPD vs NDPD, DPD vs HC, NDPD vs HC). The clusters showing significant differences (voxel-level *p* < 0.01, cluster size > 162 mm^3^ /6 voxels, corresponding to a corrected *p* < 0.05 as determined by AlphaSim correction) in ALFF between DPD and NDPD group were extracted. The mean ALFF z-values of these clusters were calculated. The pearson correlation between the mean z-values and the neuropsychological measurements was calculated using SPSS 18.0 software (SPSS, Inc., Chicago, IL).

The between-group differences in the FC were examined using post-hoc tests after the ANCOVA analysis, with age, gender, and the duration of education as covariates. The clusters showing significant differences in FC between DPD and NDPD group were extracted. The mean FC z-values of these clusters were calculated. The pearson correlation between the mean z-values and the neuropsychological measurements was calculated using SPSS 18.0 software (SPSS, Inc., Chicago, IL).

## Results

### Clinical and Demographic Testing of Sample

Twenty patients had the diagnosis of DPD according to the DSM-V criteria. There was no significant difference in age (*F* = 1.204, *P*>0.05), education years (*F* = 0.039, *P*>0.05) and gender (χ ^2^ = 2.572, *P*>0.05) among the three groups. HDRS scores were significantly different (*F* = 212.009, *P*<0.05) among the three groups. There was no significant differences in PD duration (*t* = 1.248, *P*>0.05), UPDRSIII(*t* = 0.138, *P*>0.05), H&Y (*t* = 0.718, *P*>0.05)and LED(*t* = 0.245, *P*>0.05) between NDPD and DPD groups, as shown in Table 1. Differences in HDRS scores between male and female patients were not significant in either the NDPD group (male: 6.92±3.417; female: 6.62±2.599; *P* = 0.757) or the DPD group (male: 20.56±6.044; female: 20.36±3.264; *P* = 0.933), and depression severity was not correlated with age in PD group(*r* = 0.085, *P* = 0.581).

**Table 1:** Demographic and neuropsychological characteristics of All Subjects.

Groups	HC(n = 41)	NDPD(n = 39)	DPD(n = 20)	*P* value
	Mean±SD	Mean±SD	Mean±SD	
Age(years)	56.37±5.01	54.69±10.45	58.05±7.72	0.305*
Education(years)	11.07±5.03	11.33±3.77	11.15±3.12	0.962*
Gender(M/F)	20/21	26/13	9/11	0.109#
HDRS	2.41±2.45	6.82±3.14	20.45±4.58	0.0001 a, b, c
UPDRSIII	NA	28.21±13.17	27.65±13.17	0.879⊙
H&Y	NA	1.72±0.64	1.40±0.60	0.069⊙
LED(day/mg)	NA	474.18±399.39	500.63±423.71	0.823⊙
PD duration time	NA	6.50±3.54	5.35±2.82	0.217⊙

Values are represented as the mean±SD. For comparisons of demographics:

*P values were obtained using one-way ANOVA tests;

^#^P value for the gender distribution in the three groups was obtained using a χ^2^test.

Comparisons of neuorpsychological scores among the three groups (HC, NDPD and DPD) were performed using a separate one-way ANCOVA. Post hoc test were then performed using the *t* test. The UPDRSIII, H&Y, duration and LED were compared utilizing two-sample *T*-test between NDPD and DPD for ⊙p value. NA-not applicable. F- female; M- male. P<0.05 was considered significant.

^a^ Post hoc paired comparisons showed significant group differences between HC versus NDPD.

^b^ Post hoc paired comparisons showed significant group differences between HC versus DPD.

^c^ Post hoc paired comparisons showed significant group differences between NDPD versus DPD.

### Altered ALFF in PD patients

**Fig 1:**
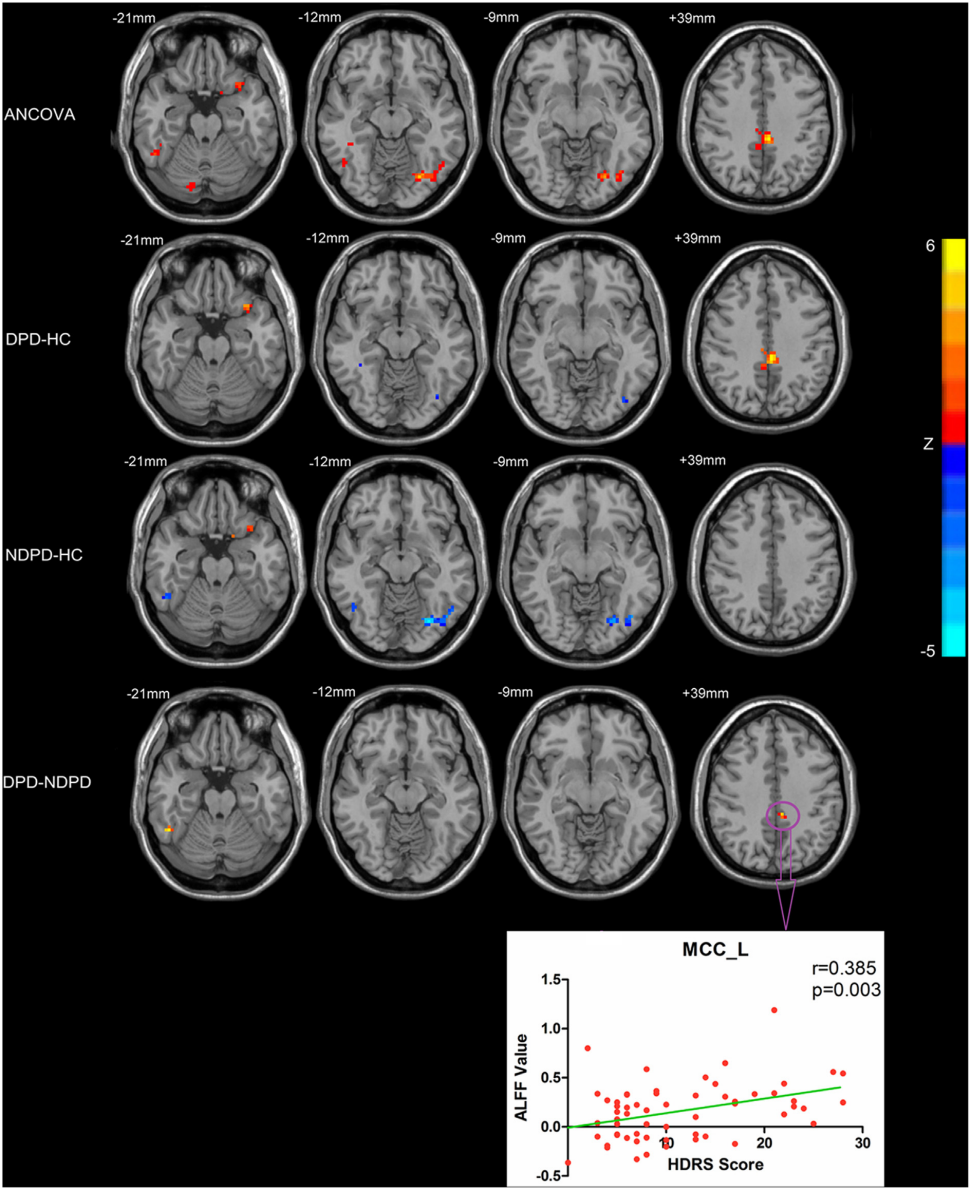
Statistical parametric map showing the significant differences in the ALFF between three groups: DPD, NDPD and HC. The ANCOVA thresholds were set at a voxel-level *p* < 0.01, cluster size > 918 mm^3^ /34 voxels, corresponding to a corrected *p* < 0.05 as determined by AlphaSim correction. A two-sample post hoc *t*-test was performed between each pair of the three groups (DPD vs NDPD, DPD vs HC, NDPD vs HC)(voxel-level *p* < 0.01, cluster size > 162 mm^3^ /6 voxels, corresponding to a corrected *p* < 0.05 as determined by AlphaSim correction). Statistical parametric map showing the significant correlations between HAMD score and ALFF value in left MCC.

An ANCOVA revealed significant differences in the ALFF among the HC, DPD, and NDPD groups in the following regions: the left inferior frontal gyrus (IFG), bilateral fusiform gyrus, right inferior temporal gyrus (ITG), and left MCC. Two-sample post hoc *t*-tests were then performed to determine differences of ALFF between each pair of the three groups (HC, DPD and NDPD) (Fig 1 and Table 2).

**Table 2:** Difference of ALFF value for All Subjects.

Brain region	Clusters Size(mm^3^)	Peak MNI			Peak Z value
		X	Y	Z	
Ancova Result					
Inferior Frontal Gyrus_L	21	-15	15	-27	12.1132
Temporal_Inf_R/ Fusiform_R	46	45	-42	-18	11.6277
Fusiform_L	33	-27	-75	-12	10.7778
Cingulum_Mid_L	34	-6	-36	39	13.4895
DPD vs HC					
Inferior Frontal Gyrus_L	12	-30	18	-21	4.6039
Fusiform_R	13	42	-36	-18	-4.0056
Fusiform_L	6	-39	-78	-9	-3.0283
Cingulum_Mid_L	34	-3	-36	39	6.0515
NDPD vs HC					
Inferior Frontal Gyrus_L	15	-15	15	-27	5.0777
Temporal_Inf_R	41	51	-51	-18	-4.1288
Fusiform_L	32	-27	-75	-12	-4.7191
DPD vs NDPD					
Temporal_Inf_R	8	51	-51	-21	3.6334
Cingulum_Mid_L	10	-6	-36	36	3.5244

Note: Pthresholds were set at a corrected P<0.01, determined by Monte Carlo simulation for multiple comparison; BA: Brodmann area; cluster size is in mm3; MNI: Montreal Neurological Institute; analysis of covariance and post-hoc two sample *T*-test with age, gender and duration of educations as covariates were performed to test the difference between the three groups in ALFF value.

Compared with the HC subjects, both the DPD and NDPD patients exhibited increased ALFF in the left IFG; and decreased ALFF in the bilateral fusiform gyrus, and these three ROIs were no significant differences between DPD and NDPD group. Compared with the DPD patients and HC, NDPD patients exhibited decreased ALFF in the right ITG, and there was no significant difference between DPD and HC group. Compared with the NDPD patients and HC, DPD patients exhibited increased ALFF in the left MCC, and there was no significant difference between NDPD and HC group (Fig 1 and Table 2).

Since the change of ALFF in the left MCC was specific to the DPD group, we hypothesized that MCC was specifically involved in the depression in PD. Hence we examined the relationship between the HDRS and ALFF values in the left MCC, and calculated the whole-brain FC with the left MCC. Results showed that ALFF values in left MCC (*r* = 0.385, *p* = 0.003) were positively correlated with the HDRS scores (Fig 1).

### Altered FC in PD patients

One-sample *t* tests were conducted on the z-maps of each of the three groups respectively. (Fig A in S1 File). ANCOVA revealed significant differences of the left MCC FC among the HC, DPD, and NDPD groups in the following regions: left cerebelum, right lingual, left IFG, left median prefrontal cortex (MPFC), right calcarine gyrus, left cuneus gyrus, left precuneus, bilateral paracentral lobule, and bilateral precentral gyrus.

**Fig 2:**
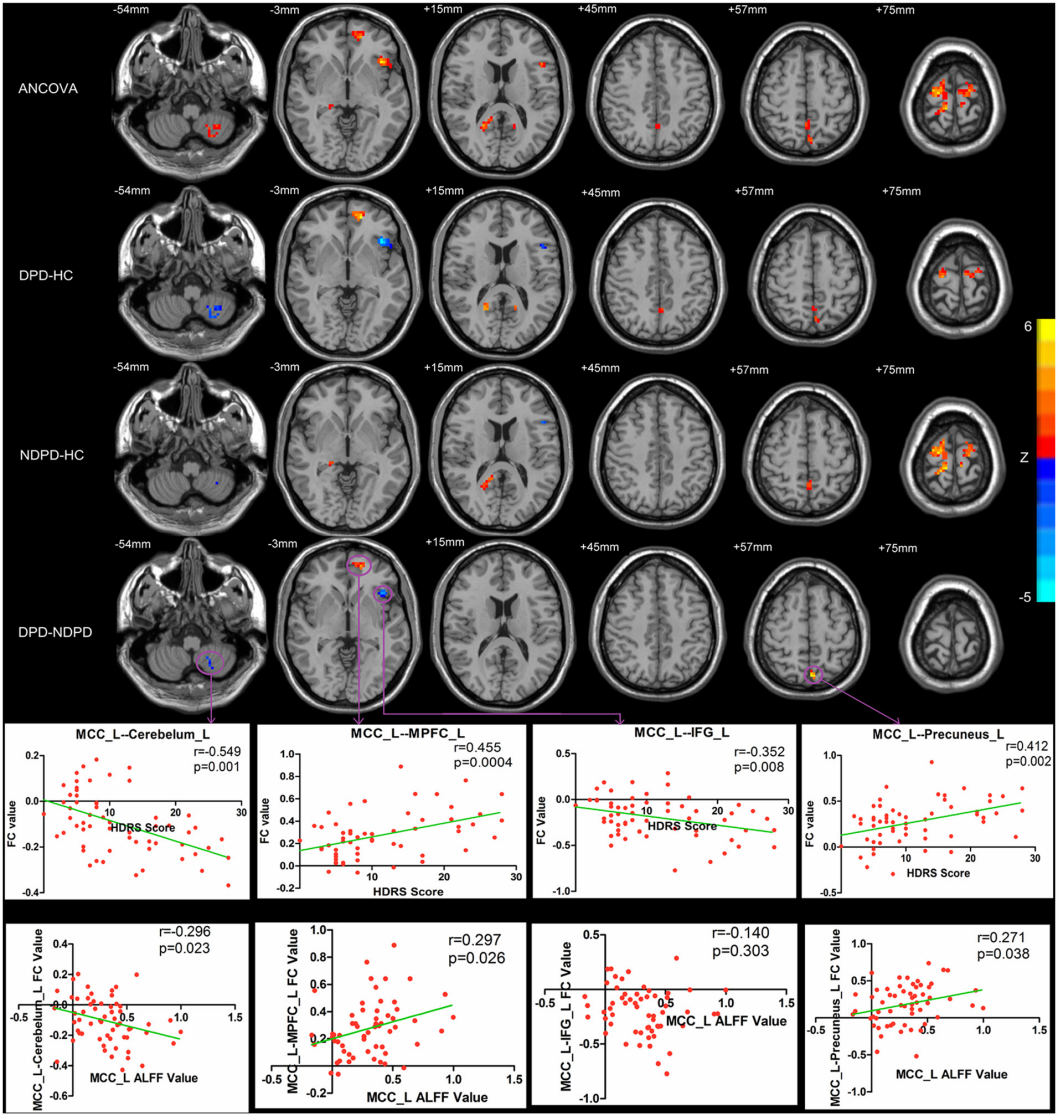
Statistical parametric map showing the significant differences in the MCC FC between three groups: DPD, NDPD and HC. The ANCOVA thresholds were set at a voxel-level *p* < 0.01, cluster size > 918 mm^3^ /34 voxels, corresponding to a corrected *p* < 0.05 as determined by AlphaSim correction. A two-sample post hoc *t*-test was performed between each pair of the three groups (DPD vs NDPD, DPD vs HC, NDPD vs HC)(voxel-level *p* < 0.01, cluster size > 162 mm^3^ /6 voxels, corresponding to a corrected *p* < 0.05 as determined by AlphaSim correction). Statistical parametric map showing the significant correlations between HAMD score and FC value in left MCC and other ROIs. Statistical parametric map also showing the significant correlations between ALFF value of left MCC and FC value in left MCC and other ROIs.

Two-sample post hoc *t*-tests were then performed to determine significant differences of left MCC FC between DPD and NDPD. Compared with the NDPD patients, DPD patients exhibited increased left MCC FC with the left precuneus and left MPFC, and decreased left MCC FC with the left IFG and left cerebellum. Compared with the HC, DPD group both showed increased left MCC FC with the left MPFC, right calcarine gyrus, left cuneus gyrus, left precuneus, bilateral paracentral lobule, and bilateral precentral gyrus, and decreased left MCC FC with left cerebellum, and left IFG. Compared with the NDPD patients and HC, DPD patients exhibited increased left MCC FC with the left precuneus and left MPFC, and decreased left MCC FC with the left IFG and left cerebellum. Compared with the HC, NDPD group showed increased left MCC FC with the right lingual, left precuneus, right calcarine gyrus, left cuneus gyrus, bilateral paracentral lobule, and bilateral precentral gyrus, and decreased left MCC with the left cerebellum and left IFG. (Fig 2 and Table 3).

**Table 3:** Difference of MCC FC value for All Subjects.

Brain region	Clusters Size(mm^3^)	Peak MNI			Peak Z value
		X	Y	Z	
Ancova Result					
Cerebellum_8_L	41	-27	-57	-51	8.7396
Cerebellum_6_L	42	-33	-69	-21	9.0824
Lingual_R	7	18	-30	-6	9.7378
Frontal_Inf_Orb_L	55	-39	21	-3	11.622
Median Prefrontal Cortex_L	28	-15	51	-3	9.1644
Frontal_Inf_Oper_L	26	-51	18	9	8.2517
Calcarine_R	12	21	-54	15	8.9655
Cuneus_L	39	-15	-66	24	9.4896
Precuneus_L	55	-3	-72	54	8.349
Paracentral_Lobule_R	20	12	-33	72	10.6411
Precentral_R	25	18	-15	75	12.7124
Paracentral_Lobule_L/ Precentral_L	24	-18	-12	75	8.9361
DPD vs HC					
Cerebellum_8_L	27	-21	-57	-54	-3.7182
Cerebellum_6_L	35	-36	-69	-21	-3.913
Frontal_Inf_Orb_L	45	-39	21	-6	-5.4857
Median Prefrontal Cortex_L	28	-15	51	3	4.3857
Frontal_Inf_Oper_L	25	-48	12	9	-3.5177
Calcarine_R	10	21	-54	15	3.8028
Cuneus_L	32	-12	-66	21	3.6799
Precuneus_L	44	-3	-69	54	3.5436
Precentral_R	15	21	-18	75	3.6573
Paracentral_Lobule_L/ Precentral_L	14	-6	-15	75	3.2991
NDPD vs HC					
Cerebellum_8_L	12	-33	-57	-48	-3.5299
Cerebellum_6_L	24	-27	-69	-24	-3.6791
Lingual_R	7	18	-30	-6	4.7404
Frontal_Inf_Orb_L	12	-51	18	9	-3.9228
Calcarine_R	12	21	-57	21	3.6225
Cuneus_L	21	-15	-66	24	3.4642
Paracentral_Lobule_R	20	9	-33	75	4.5725
Precentral_R	22	18	-15	75	4.7791
Precentral_L	15	-18	-18	75	3.7611
Paracentral_Lobule_L	8	-9	-30	75	3.7189
Precuneus_L	8	-3	-51	60	3.2989
DPD vs NDPD					
Cerebellum_8_L	17	-18	-60	-45	-3.9512
Frontal_Inf_Orb_L	16	-39	21	-3	-3.5237
Median Prefrontal Cortex_L	19	-15	54	-3	3.548
Precuneus_L	18	-6	-72	57	4.3114

Note: Pthresholds were set at a corrected P<0.01, determined by Monte Carlo simulation for multiple comparison; BA: Brodmann area; cluster size is in mm3; MNI: Montreal Neurological Institute; analysis of covariance and post-hoc two sample *T*-test with age, gender and duration of educations as covariates were performed to test the difference between the three groups in FC value.

Further analysis revealed that FC values in the four regions were significantly correlated with the HDRS scores, including the FC values between the following pairs: the left MCC—left precuneus (*r* = 0.412, *P* = 0.002), the left MCC–left MPFC (*r* = 0.455, *P* = 0.0004), the left MCC—left IFG (*r* = -0.352, *P* = 0.008), and the left MCC—left cerebellum (*r* = -0.549, *P* = 0.001). The ALFF values in the MCC were also significantly correlated with the FC values between the MCC and the following areas: left precuneus (*r* = 0.271, *P* = 0.038), the left MPFC (*r* = 0.297, *P* = 0.026), and the left cerebellum (*r* = -0.296, *P* = 0.023) (Fig 2).

## Discussion

We explored the association between depressive symptoms and abnormal brain activities, and functional networks in PD using RS-fMRI. We found that DPD patients exhibited increased activity in left MCC and altered FC in the limbic-parietal, limbic-frontal and limbic-cerebellum circuits.

In our current study, we have found increased local activity in the left MCC in the DPD patients compared with both the NDPD and HC groups. Further analysis revealed that ALFF values in the left MCC was significantly positively correlated with the HDRS scores. The cingulate cortex plays key roles in integrating multimodal information important for emotional, sensorimotor, and cognitive functions[18], and the MCC appears to be involved in many emotional-related cognitive processes such as meditation, self-related rumination, aversive conditioning, and the anticipation and perception of pain[19]. Various fMRI and Positron Emission Computed Tomography studies have found increased resting-state BOLD activities or regional cerebral metabolic rates for glucose in the MCC of patients with major depressive disorder (MDD)[20] or posttraumatic stress disorder[21]. Our results are consistent with these studies, the hypermetabolism in the MCC in the DPD may indicate increased negative rumination or more frequently emerged depressive feelings.

Compared with NDPD group, we found increased activity in right ITG in DPD group, but the activity of right ITG was not significant between DPD and HC. Compared with NDPD group, the activity in right ITG also increased in HC group. Previous studies suggested that the temporal lobe was involved in emotional processing[22, 23], and decreased activities of the temporal lobe in MDD were also reported[24]. Yang et al. [25] have found increased activities in the temporal lobe in early PD patients. Since NDPD patients showed decreased activities in the ITG, we speculated that ITG might be associated with the comorbidity of motor defects and depression rather than being simply specific to depression, and that these two aspects may influence separate as well as overlapping the temporal lobe. We also performed the ITG FC as a supplement material (Fig B in S1 File).

Previous studies [25–27] have reported damaged microstructural integrity and abnormal activities in the IFG, and fusiform gyrus in PD patients. Here, our study also showed PD patients had increased ALFF in the left IFG and decreased ALFF in the bilateral fusiform relative to the HC, but there were no significant difference between DPD and NDPD group. Since these changes were same in the DPD and NDPD groups rather than specific to the DPD group, they might not be depression-specific markers in PD.

Network level analysis showed stronger FC between the MCC and the MPFC, precuneus, cerebellum, and IFG in the DPD relative to the NDPD. Further analysis revealed these FC values between the left MCC and these ROIs were significantly correlated with the HDRS scores. Previous neuroimaging studies have also found abnormal changes in these regions in PD patients[25, 28, 29], however whether these changes were due to motor symptoms or other comorbid psychiatric symptoms was not elucidated. Here we thought that the altered FC between the MCC and these regions were correlated with depression in PD. These cortical areas are important components of the DMN and are responsible for high-order cognitive control and emotion regulations, and dysfunctions of these regions and enhanced FC within the DMN have been observed in MDD [2, 30–32]. Increased local activities of the MCC were correlated with enhanced FC between the MCC and the DMN nodes in the current study, implying that local abnormalities and hyperfunctioning of the MCC might have led to network level abnormalities in the limbic-cortical circuits. The limbic areas may impose stronger influence on the cortical areas through enhanced FC and impair the ability of emotion regulations and increase the sensitivity to a range of negative emotions in DPD patients[20]. Our findings suggested that these cortical structures form interconnecting circuits might be more vulnerable to negative emotions emerged from the subcortical limbic structures in DPD patients, as has been suggested in the limbic-cortical dysregulation model of MDD[20].

The present research was a preliminary study, some technical and biological limitations inevitably exist. First, our study was a cross-sectional study with a relatively small DPD sample size, and number of the subjects in the three groups did not match perfectly. To control for the effects of the differences in age, gender and education among the three groups, these variables were considered as covariates and regressed out in the statistical analysis. Second, the patients in the current study were not drug naive. Current and chronic medication is often a potential confound and few trials have examined the immediate or delayed effects of L-dopa on resting-state brain activities. Luo et al[14] have reported the DPD group showed significantly higher ALFF in the left orbitofrontal area compared with both the NDPD and HC groups, whereas we found increased ALFF in the left orbitofrontal areas in both DPD and NDPD groups. Since the patients were drug naïve in Luo’s study, the use of L-dopa drugs in the patients in the current study may explain the differences between their results and ours.

## Conclusion

In the current study, we found both local and global level abnormal changes in the DPD patients, these changes were correlated with the depression in PD. Local abnormalities in the MCC might impose stronger influence on the DMN areas through enhanced FC and lead to network level abnormalities in the limbic-cortical circuit. Our results indicate that the neural mechanisms of the development of depression in PD might be similar to the limbic-cortical dysregulation model in MDD.

## Supporting Information

S1 File Statistical parametric map showing the one sample *T*-test result in the MCC FC in the three groups: DPD, NDPD and HC. The one sample *T*-test thresholds were set at a voxel-level *p* < 0.01, cluster size > 918 mm^3^ /34 voxels, corresponding to a corrected *p* < 0.05 as determined by AlphaSim correction (**Fig A**). Statistical parametric map showing the one sample *T*-test result in the ITG FC in the three groups: DPD, NDPD and HC. The one sample *T*-test thresholds were set at a voxel-level *p* < 0.01, cluster size > 918 mm^3^ /34 voxels, corresponding to a corrected *p* < 0.05 as determined by AlphaSim correction. A two-sample post hoc *t*-test was performed between each pair of the three groups (DPD vs NDPD, DPD vs HC, NDPD vs HC)(voxel-level *p* < 0.01, cluster size > 162 mm^3^ /6 voxels, corresponding to a corrected *p* < 0.05 as determined by AlphaSim correction)(**Fig B**).
(ZIP)
