# Supplementary figures and images for: Altered Resting-State Brain Activity and Connectivity in Depressed Parkinson’s Disease
Source: PLoS One. 2015 Jul 6;10(7):e0131133. doi: 10.1371/journal.pone.0131133 (PMC4492789; doi:10.1371/journal.pone.0131133)

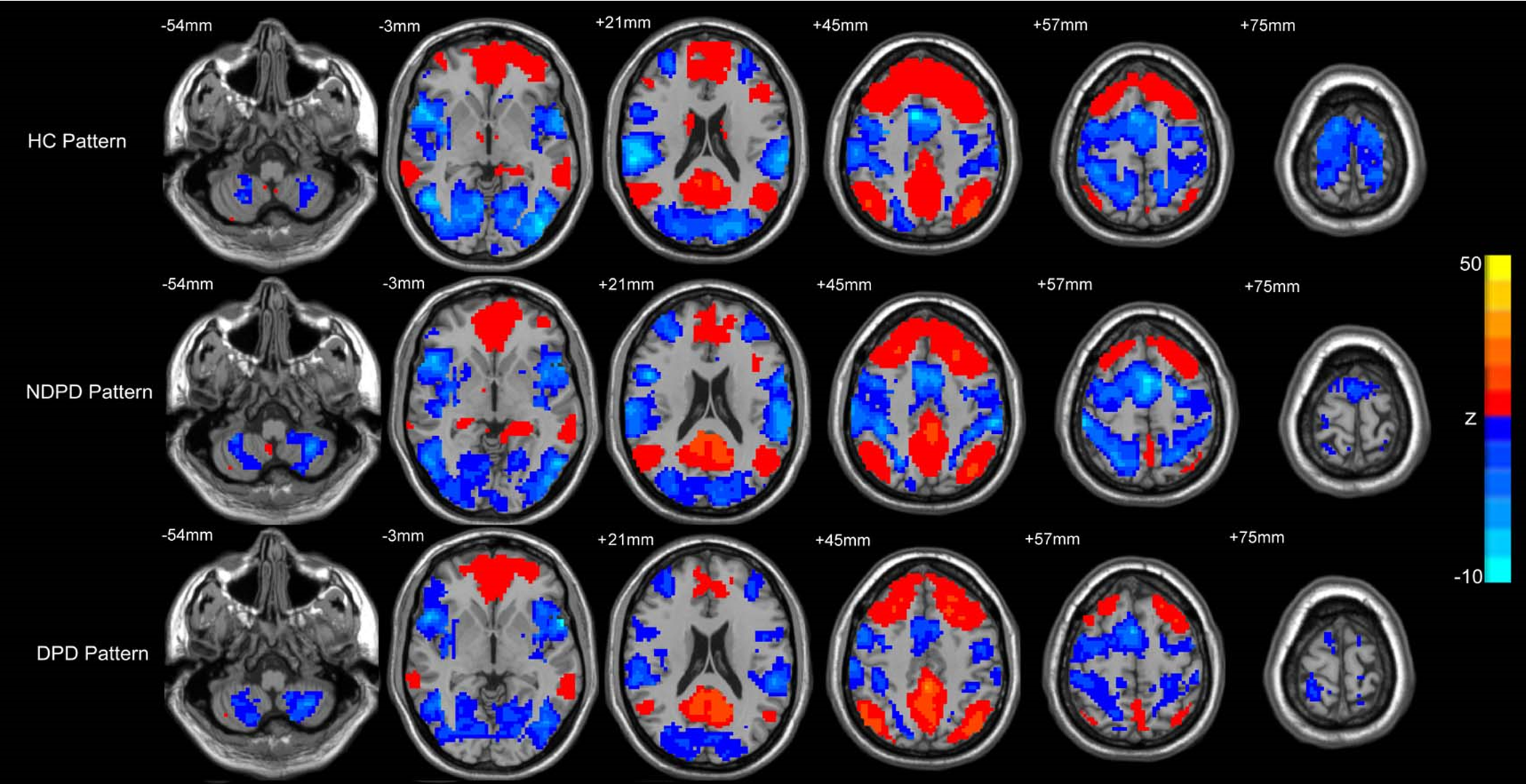

Supplement: S1 File — The one sample T-test thresholds were set at a voxel-level p < 0.01, cluster size > 918 mm3 /34 voxels, corresponding to a corrected p < 0.05 as determined by AlphaSim correction (Fig A). Statistical parametric map showing the one sample T-test result in the ITG FC in the three groups: DPD, NDPD and HC. The one sample T-test thresholds were set at a voxel-level p < 0.01, cluster size > 918 mm3 /34 voxels, corresponding to a corrected p < 0.05 as determined by AlphaSim correction. A two-sample post hoc t-test was performed between each pair of the three groups (DPD vs NDPD, DPD vs HC, NDPD vs HC)(voxel-level p < 0.01, cluster size > 162 mm3 /6 voxels, corresponding to a corrected p < 0.05 as determined by AlphaSim correction)(Fig B). (ZIP) [file pone.0131133.s001.zip › S1 File/Fig. A.tif]

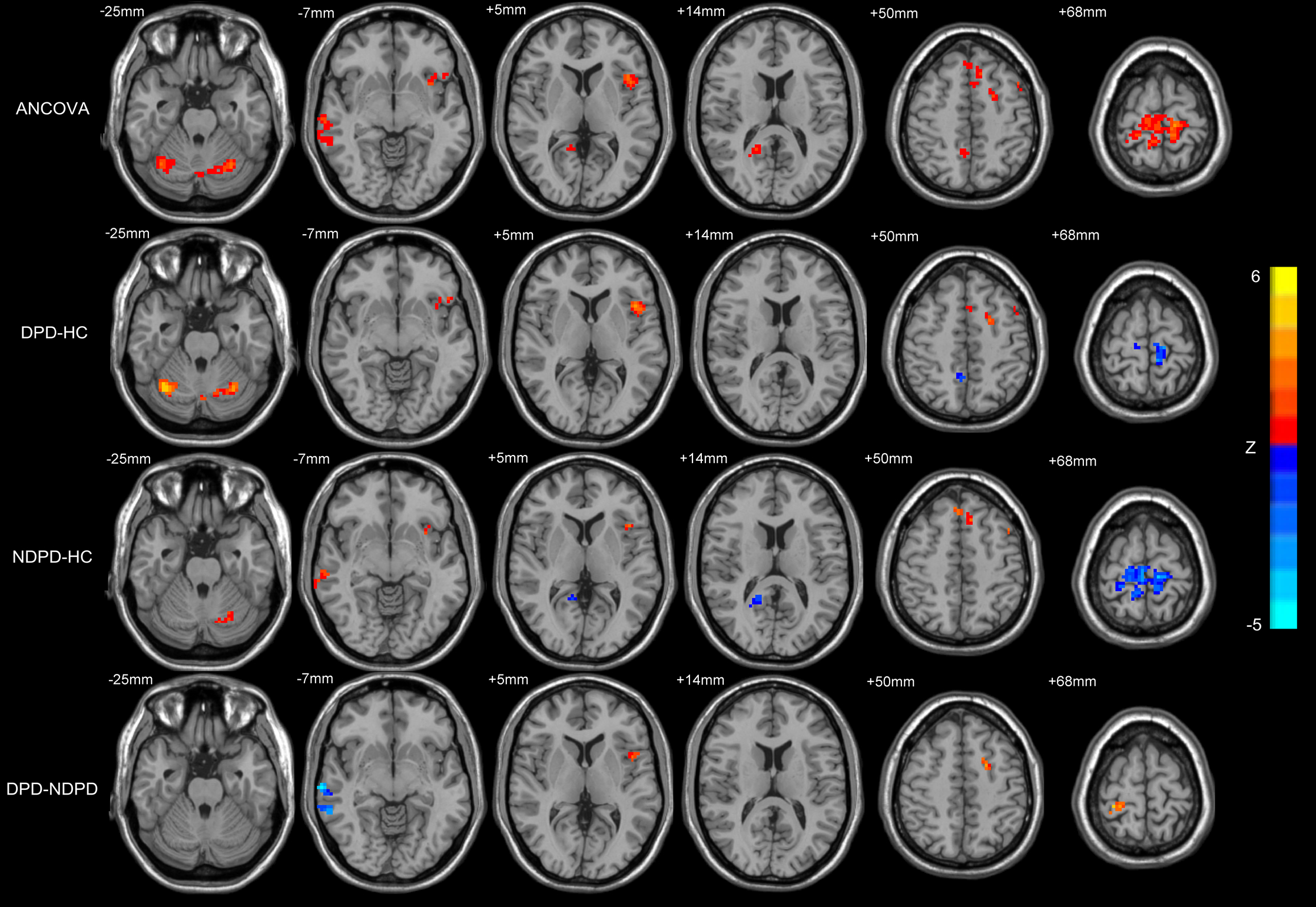

Supplement: S1 File — The one sample T-test thresholds were set at a voxel-level p < 0.01, cluster size > 918 mm3 /34 voxels, corresponding to a corrected p < 0.05 as determined by AlphaSim correction (Fig A). Statistical parametric map showing the one sample T-test result in the ITG FC in the three groups: DPD, NDPD and HC. The one sample T-test thresholds were set at a voxel-level p < 0.01, cluster size > 918 mm3 /34 voxels, corresponding to a corrected p < 0.05 as determined by AlphaSim correction. A two-sample post hoc t-test was performed between each pair of the three groups (DPD vs NDPD, DPD vs HC, NDPD vs HC)(voxel-level p < 0.01, cluster size > 162 mm3 /6 voxels, corresponding to a corrected p < 0.05 as determined by AlphaSim correction)(Fig B). (ZIP) [file pone.0131133.s001.zip › S1 File/Fig. B.tif]
